# Factors associated with scientific production citations in dentistry: Zero-inflated negative binomial regression and hurdle modelling

**DOI:** 10.12688/f1000research.141422.1

**Published:** 2023-10-12

**Authors:** Pablo Alejandro Millones-Gómez, Carlos Alberto Minchón-Medina, David Yeret Rodríguez-Salazar, Jorge Gustavo Alonso Delgado-Caramutti, Alejandro Valencia-Arias

**Affiliations:** 1 Universidad Senor de Sipan, Chiclayo, Lambayeque, Peru; 2 Universidad Nacional de Trujillo, Trujillo, Peru

**Keywords:** bibliometric, dentistry, scientific production, pandemic, infection control, Dentistry, Research methods, Information / Knowledge Management

## Abstract

**Background:** The global scientific literature in dentistry has shown important advances in the field, with major contributions ranging from the analysis of the basic epidemiological aspects of prevention to specialised results in the field of dental treatments. The present investigation aimed to analyse the current state of the scientific literature on dentistry hosted in the Web of Science database.

**Methods:** The methodology included two phases in the analysis of articles and indexed reviews in all thematic areas. During the first phase, the following variables were analysed: scientific production by the publisher, the evolution of scientific output published by publishers, the factors associated with the impact of scientific production, and the modelling of the impact of scientific production on dentistry. During the second phase, associations, evolutions, and trends in the use of keywords in the scientific literature in dentistry were analysed.

**Results:** The first phase shows that scientific production in dentistry will increase between 2010 and 2021, reaching 12,126 articles in 2021. Publishers such as Wiley and Elsevier stand out, but Quintessence Publishing has the most citations. Factors such as pages, authors, and references influence the number of citations. Phase 2 analyzes trends in the dental literature using the WoS database. Topics such as “dental education”, “pediatric dentistry”, and “pandemic” stand out. The intersection of technology and dentistry and the importance of evidence-based education are highlighted.

**Conclusions:** In conclusion, the study shows that the most studied topics include the association of dental education and the curriculum, the association of pediatric dentistry with oral health, and dental care. The findings show that more recently emphasised topics also stand out, such as evidence-based dentistry, the COVID-19 pandemic, infection control, and endodontics, as well as the need for future research to expand current knowledge based on emerging topics in the scientific literature on dentistry.

## Introduction

Scientific or research activity has often been evaluated based on different bibliometric indicators. Bibliometrics is a set of mathematical and statistical methods used to analyse and measure the number and quality of books, articles, and other forms of publications. Through bibliometrics, we can gain an understanding of scientific productivity and thus quantify scientific production; additionally, we can recognise which authors, journals, institutions, or even countries are most interested in the generation of knowledge about a topic. ^1^ Quality indicators allow for the evaluation of the impact of researchers, institutions, or the scientific community, for example journal impact factors, crown indicators, and the hindex (or Hirsch index) and its variants. ^2^^,^^3^ In addition to indicators measuring volume and quality, some bibliometric analyses also examine the structure that, according to García-Villar and García-Santos, ^3^ allows for an appreciation of different aspects of scientific cooperation and thematic alliance. These indicators facilitate a more holistic, detailed, and useful understanding of the scientific information housed in specialised databases. ^4^

This analysis encompasses not only the visibility, relevance, and impact of the articles but also considers aspects of prestige. ^5^ These standardised indicators in all disciplines, for which there is great interest, ^6^ are calculated by using the data available in international databases, ^7^ thus addressing some research gaps. As the current scientific community has become increasingly competitive and global, and as digital technology has emerged in conjunction with the rapid evolution of journals acting as platforms for the dissemination of data and other digital objects, ^8^ editorial excellence has become an overarching goal. ^9^

The creation of new indicators has been particularly important as journals are evolving rapidly and becoming platforms for the dissemination of data, methods, and other digital objects. The quality of the articles submitted to dental and scientific journals, plagiarism, attempted duplication, and sometimes fraudulent results are all challenges publishers and publishing companies must face. ^10^ In addition, one of the main challenges for the editors of scientific journals is the analysis of self-citations, which also affects aspects associated with scientific impact. ^11^ Hence, it is important for publishers to understand scientific production and its impact.

A series of factors are associated with problems in scientific production and its impact, including the lack of resources for developing research, poor preparation of the manuscripts sent to journals, limited access to quality literature in scientific databases, conflicts in relation to publication, and limitations in the journal review process; therefore, it is necessary to be aware of these limitations and to monitor them to achieve a better representation in the thematic areas of scientific journals. ^12^^–^^15^

A review of research indexed in some search engines such as PubMed reveals that the number of publications has doubled over the last two decades globally, as evidenced by Godín. ^4^ The reach of a research field, the extent to which publications are concentrated, and the characteristics of citations are fundamental considerations in carrying out bibliometric analyses. In addition, factors such as variations in the frequency of publication of articles by topic indicate different research priorities at a global level, as in the case of research on epidemiological trends and changes in the practice of dentistry. ^16^ Over the last 30 years, scientific production in dentistry, while maintaining a similar structure in terms of the represented specialties, has varied both in terms of qualitative and quantitative research models and in its geographical distribution ^17^; moreover, it has been recognised that these factors are distinctly important for the advancement of science and that a small number of studies stand out. ^18^

The increase in the number of scientific publications is an important aspect to consider in bibliometric analyses in various fields, such as dentistry. In some cases, these analyses have only described trends. ^19^^,^^20^ Indeed, some analyses have indicated that there were trends ^21^; that there was no clear trend ^22^; that the increase in the number of publications was linear, ^23^ polynomial, ^24^ or exponential ^25^; or that its trends followed a discontinuous regression pattern. ^26^ This information will guide researchers in dentistry when they make decisions about which journal to publish in and therefore to which publisher they should submit their article.

The number of times scientific papers are cited in other studies is a reflection of their quality. However, this factor does not distinguish whether the citation has a positive or critical connotation. ^27^ In addition, some authors point out that the number of citations should not be considered a sufficiently nuanced or solid quality measure if it is used in isolation. ^28^

Scientometrics experts analyze the reasons for the lack of citations in certain articles. Articles without citations are typically authored by a single individual, which may suggest that they offer less comprehensive information. These articles tend to have fewer references and are generally shorter in length. ^29^

The analysis of the number of citations or noncitations for articles published in journals around the world involves the consideration of important aspects. First, factors that affect the highest number of citations must be considered. In dentistry, various factors have been studied: years since publication, ^30^^,^^31^ number of pages, ^30^ type of article, ^29^^,^^32^ topic covered in the article, ^30^^,^^33^ journal, ^2^^,^^31^^–^^33^ number of authors, ^2^^,^^33^ and number of references, ^2^ among others. Second, it is concerning for researchers and journals not to be cited, and there are various reasons why an article is not cited, for instance, the article’s topic might be highly specialized and irrelevant to current research, or the distribution and accessibility of the publication might be restricted, hindering other researchers from discovering and citing it as a reference. ^34^ It has been recommended that noncitation rates should be made available for all journals. ^35^ These factors are of interest for the present study.

A third factor has been considered to explore the question of citations. In dentistry, linear regression models, ^20^ Poisson regression models, ^31^ and negative binomial regression models ^2^^,^^32^^,^^33^ have been used to identify the factors associated with the number of citations; in particular, negative binomial regression models have been used in the presence of overdispersion in the number of citations. ^34^ However, these models, by themselves, do not allow the study of the factors impacting the number of citations and noncitations, whereas zero-inflated negative binomial regression models and double fence models do have this capability as part of bibliometric ^36^ or computer science ^37^ studies.

Hence, the present investigation aimed to analyse the current state of the scientific literature on dentistry hosted on the Web of Science (WoS) database. The models’ scientific production in journals is classified by publisher using discontinuous regression models. The factors associated with citations and noncitations of scientific articles in dentistry published in the WoS are determined using zero-inflated negative binomial regression models and double fence models. In addition, a differentiated analysis of the main keywords is carried out to account for the activities taking place in the field and the thematic evolution of that field.

In this study, some research questions were formulated to achieve objectives and guide a discussion. The research questions are as follows:

RQ1: What statistical model does scientific production on dentistry follow in the main publishing houses?

RQ2: What are the factors associated with the citation of scientific production in dentistry?

RQ3: What are the main clusters that define conceptual associativity in dentistry?

RQ4: How have themes covered in the scientific literature on dentistry evolved?

RQ5: What keywords have emerged and been increasingly used in the dentistry scientific literature?

## Methods

The present study is based on a bibliometric-computer analysis ^36^^,^^37^ of scientific articles in dentistry during the period 2010-2022 that were published in journals across the world and extracted from the WoS database. The analysis includes the evolution of scientific production over time, the factors associated with articles in dentistry being cited and not cited, and regression models for the selection of factors.

### Data collection

The data for the present investigation were retrieved from the WoS database in August 2022; the search criteria was “dentistry”, and the search was limited to the latest articles published in journals. The total number of articles was 110,269, with 9,034 published in 2022. The files were downloaded in Excel to extract study variables such as the total number of citations (TCitations), number of pages (NPages), year of publication (Year), number of years between the time of publication and 2022 (PYears), number of authors (Nauthors), and number of references (Nreferences); the journal’s publisher was also downloaded as a variable. The name of the publisher was recorded if it was one of the top 10 in a list that was developed later, and the rest were classified into the “other” category.

The bibliometric information downloaded contained the study variables, but it was necessary to resort to text separation functions with the Excel function “Text in columns” to quantify these variables. The work was carried out by two specialists of regression models.

### Data analysis

To analyse the obtained information, the methodological design involved two phases: first, statistics were used to evaluate scientific production and extract important indicators, such as the main publishers, the annual production, as well as evaluate the scientific impact based on the previously obtained variables.

The annual scientific production on dentistry in the WoS between 2010 and 2022 was used to unveil the top 10 publishers, whose average production from 2010 to 2021 was compared using the analysis of variance (ANOVA) and Tukey’s test in the Statistical Package for Social Sciences (SPSS) in version 28. Likewise, for the same period, the annual trend in the number of publications on dentistry was evaluated using discontinuity regression models, a method applied to dental visit copayments. ^38^ These models showed that scientific production increased in any given year with some discontinuity in the trend. When the associated parameter was not significant, it was reduced to a regression model by sections. The modelling was performed in Excel.

The impact of the publications was measured by the number of citations the articles received or by the occurrences of noncitation. The program used to select these factors was Stata 16, and the z test was used to count the models with the significance set at p < 0.05. The analysis of the impact included 76,641 of the 110,269 articles, which were downloaded for the period from 2010 to August 2022 and covered the 10 publishers in the ranking. The program used to estimate the models was Stata 16. Overdispersion in the Poisson model was evaluated using the Lagrange multiplier test; moreover, the standard negative binomial regression model was evaluated through the likelihood ratio (LR) test for the α parameter (if α = 0, there is no overdispersion). ^39^

The counting models used were the zero-inflated negative binomial regression model and the hurdle model, which allowed us to analyse both the number of citations and noncitations for each article. ^36^^,^^37^ The dependent variables were the total number of citations and noncitations per article, and the independent variables were the number of pages, year of publication, number of authors, and number of references in each article. The editors working within the top 10 journals in which the articles were published constituted a categorical independent variable, and the BioMed Central (BMC) editorial board was used as a reference. The choice of a zero-inflated model versus a standard model was made using the Vuong test, and the Akaike criteria (AIC) and Bayesian (BIC) approach were used to choose the best model. ^39^

Subsequently, in the methodological design, there was a second phase of bibliometric analysis, and once the statistical analysis of the main journals was advanced, the evaluation of the impact and of the annual scientific productivity focused mainly on the materialisation of the analysis of the keywords derived from the scientific activity.

During this process, an exclusion criterion that allows the content analysis of the main scientific articles that are available in the field was chosen so that, of the total number of articles selected for the first phase in the methodological design, 1,000 articles were used as a filter. Moreover, at the time the present research was conducted, these articles accounted for the highest total number of citations and were considered the main references in the field.

Once the extraction of the thematic analysis of the main articles was carried out, the main network was analysed to determine the co-occurrence of keywords, allowing for an understanding of the elements of thematic associativity. We also conducted an analysis of the validity and frequency of the keywords so that consolidated or increasingly used keywords were extracted as well as the keywords emerging within the research field. Finally, an analysis of the thematic evolution was carried out based on the main keywords for each research year based on the selected articles.

**Figure 1.**
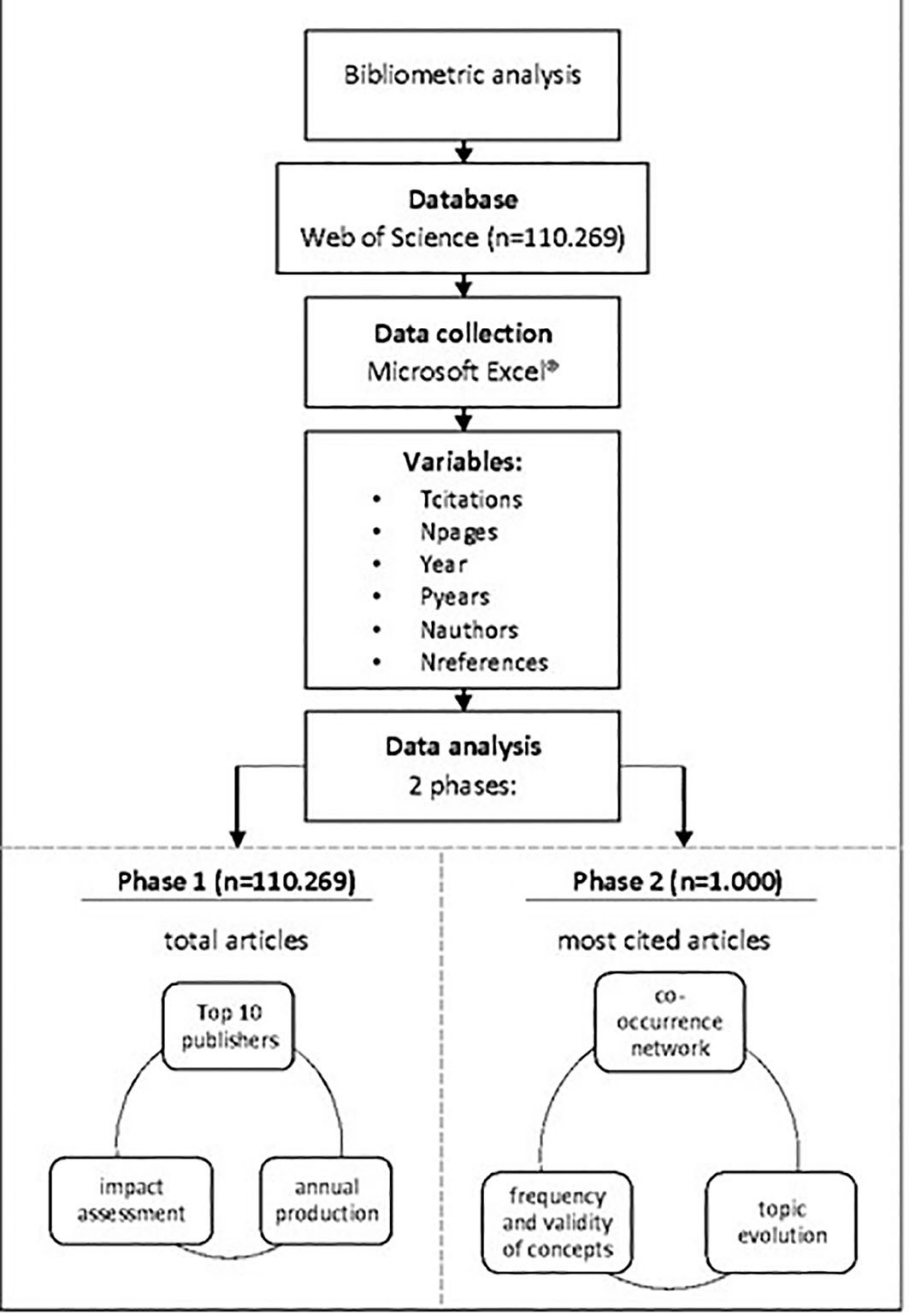
Summary of the methodological design used in this research. Tcitations: total of citations, Npages: number of pages, Pyears: publication years, Nauthors: number of authors, Nreferences: number of references. Figure 1 Alt Text. The figure provides a concise overview of the methodological design employed in the research, encompassing the analysis of co-occurring keywords using VOSviewer, tracking emerging words and thematic evolution through Microsoft Excel®, and collaborative author involvement for minimizing biases and achieving consensus.

To execute the analysis of the main network and determine the co-occurrence of keywords in the scientific body, the free access software VOSviewer was used, while the analysis of the increasingly used and emerging words as well as the thematic evolution was carried out using the office automation tool Microsoft Excel. This phase in the methodological design was completed collectively by all of the authors of the present research with the purpose of reducing biases in the results and jointly treating the disagreements found until convergence was attained. To increase the level of replicability and further detail the methodology, Figure 1 presents a summary of the methodological design used in the research.

## Results

### Phase 1 – Statistical evaluation of scientific production

In accordance with the nature of the methodological design, Phase 1 of the results is presented in this section. This phase involved a rigorous analysis of the scientific output published by each publisher as well as this output evolved within the time window selected for the extraction of articles. The analysis also included the factors associated with the impact of scientific output.

### Evolution of scientific production by publisher

**Figure 2.**
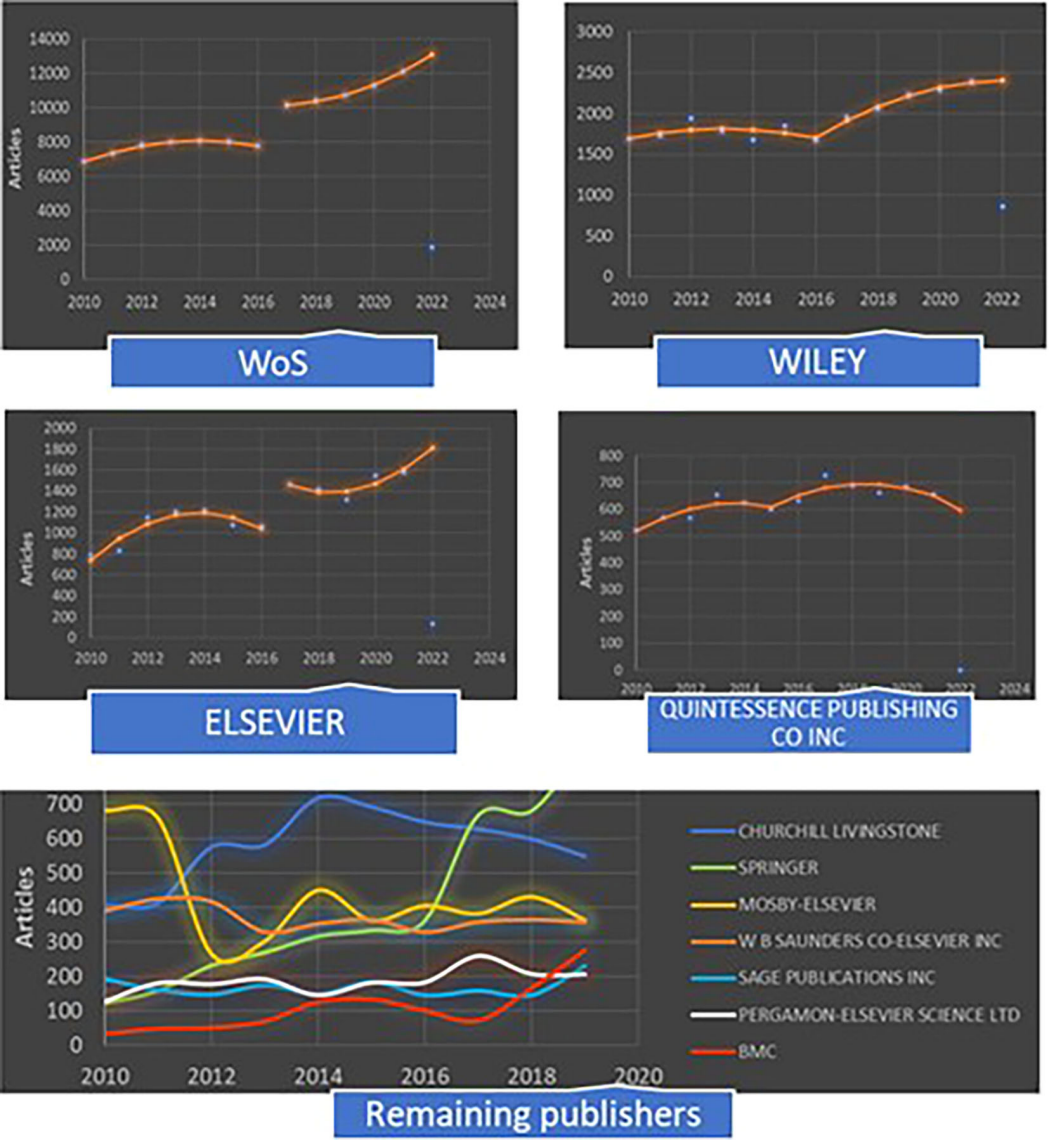
Discontinuity regression models for scientific production. Source: Published by publishers in the field of dentistry. Figure 2 Alt Text. Figure illustrates the scientific production trend in dentistry from 2010 to 2021 using polynomial models of discontinuous regression and regression models for the top 10 main publishers.

Figure 2 shows the evolution of scientific production in dentistry during the period 2010-2021 through polynomial models of discontinuous regression and regression models according to the selection in the top 10 main publishers.

The WoS database is a platform composed of a large collection of databases from all disciplines of knowledge organised by the British-American company Clarivate Analytics. ^40^^,^^41^ In 2016, the scientific production in the WoS changed to an estimated 2,377 articles (t = 14.1, p = 0.000). During the first period—2010-2016—the growth curve was concave, with the number of articles barely exceeding 8,000 per year; however, the growth curve was convex for the period between 2017 and 2021, with more than 10,000 articles/year and R2 = 99.9%. The scientific citation indexing service is based on the online subscription Book Citation Index (BKCI), which is part of WoS and is maintained by Clarivate Analytics. The BKCI provides analytic services for academic and scientific research data and was, in a limited manner, part of the WoS Core Collection for 14 years until 2017. Since 2018, it has become a perpetually maintained database. ^42^ In 2018, the complementary tool Kopernio, an extension for browsers that saves time and allows direct access to the best articles available in PDF format, was acquired by Clarivate Analytics, revolutionising access to articles from anywhere in the world. ^43^

The curves corresponding to Wiley’s activities are concave in each section. In the first section, the production is practically stable without a linear trend (p = 0.211 > 0.05) or quadratic trend (p = 0.211 > 0.05). However, in the second section, the linear trend increases (p = 0.029 < 0.05), and the quadratic trend is not evident (p = 0.690 > 0.05) and R2 = 92.7%.

Like that of the WoS, the curve corresponding to Elsevier’s activities is concave and convex, showing a discontinuity of 550 articles/year (t = 2.85, p = 0.029) and R2 = 94.3%. The activities of Quintessence Publishing are shown to have slightly changed during the 2015-2022 period compared to the 2010-2014 period, with R2 = 83.8%. In this regard, a linear model would have presented an increasing annual trend of approximately 13 articles/year (t = 4.11, p = 0.002 < 0.05) and R2 = 62.9%.

The regression models for scientific production published in the WoS and Elsevier are discontinuous regression models with leaps in 2017, and those corresponding to Wiley and Quintessence Publishing are reduced to regression models by sections.

The curve for the Churchill Livingstone publishing house shows a difference in the number of publications between the periods 2010-2014 and 2015-2021, ascending in the first and descending in the second. The number of publications by Springer Nature can be divided into the 2010-2016 and 2017-2021 periods; both periods exhibit positive growth in those numbers but changes are more intense in the second period. The number of articles published by MOSBY-ELSEVIER declines in 2012 and experiences a slow recovery thereafter. Activities in the other top 10 publishers fluctuate, with a stable number of publications or small increases throughout the study period. It is important to note that the number of articles to be published in 2022 by these publishers is predicted to be lower than expected, as shown in the trend analysis. The models used for the number of publications produced by these publishers were not estimated.

### Scientific output published by publishers

The annual scientific production on dentistry published in journals made up the top 10 categories during the period 2010-2022, as shown in Table 1. The number of published articles increased from 6,850 in 2010 to 12,126 in 2021 at a rate of 440 articles/year, with great changes between 2016 and 2017, when the number of published articles reached 2,330 articles over one year. The trend in the number of articles published by publishers, in some cases, shows very similar patterns. As of 2022, some journals still had not published related publications.

**Table 1.** Scientific output in journals published by the top 10 publishers in dentistry.

Publisher	2010	2011	2012	2013	2014	2015	2016	2017	2018	2019	2020	2021	2022	Total	Mean (2010-2021)
WILEY	1685	1724	1943	1779	1674	1847	1669	1965	2057	2234	2282	2401	865	24125	1938
ELSEVIER	791	830	1145	1207	1218	1072	1069	1461	1416	1319	1550	1577	135	14790	1221
QUINTESSENCE PUBLISHING CO INC	525	569	567	654	625	599	633	726	688	662	687	657	0	7592	633
CHURCHILL LIVINGSTONE	407	414	576	582	718	692	648	628	599	549	528	478	0	6819	568
SPRINGER	121	158	231	269	316	333	361	667	680	866	947	1338	454	6741	524
MOSBY-ELSEVIER	683	659	265	302	450	362	405	382	431	363	435	459	0	5196	433
WB SAUNDER S CO-EL SEVIER ING	391	427	419	330	354	366	330	359	366	359	322	309	25	4357	361
SAGE PUBLICATIONS ING	194	162	148	175	149	186	146	159	146	232	266	458	235	2656	202
PERGAMON-ELSEVIER SCIENCE LTD	128	181	178	192	146	182	183	259	207	206	232	168	14	2276	189
BMC	35	50	53	71	127	135	102	73	167	276	341	659	0	2089	174
Others	1890	2132	2307	2443	2242	2255	2223	3420	3675	3634	3651	3622	134	33628	2791
Total	6850	7306	7832	8004	8019	8029	7769	10099	10432	10700	11241	12126	1862	110269	9034

The quantity of published journal articles is merely one of numerous factors taken into account when assessing publishing companies such as Wiley in rankings, as illustrated in this study. This parameter, while vital, is only a component of a larger set of metrics and criteria utilized to evaluate the academic and scientific significance, influence, and importance of publishers. Publishers strive to enhance their image by increasing the number of publications and ensuring their quality and relevance. Besides, several other factors, such as article citation frequency, impact on the scientific community, and journal accessibility, also determine their rankings.

### Factors associated with the impact of scientific output

In general, the average number of citations per article was 13.4 ± 23.79, with a maximum of 1,621 citations; noncitations reached 12.08%. On the one hand, citations are the basis of the SCImago Journal Rank (SJR) index that ranks journals, and noncitations also represent an indicator for the quality of the researchers, articles, journals, and now publishers. On the other hand, the average number of pages was 7.61 ± 2.84, the average number of authors was 5.19 ± 2.59, the average number of references was 31.63 ± 16.15. These results are also important for authors to consider when producing scientific articles in dentistry.

The number of citations for the articles published in journals owned by the top 10 publishers and the factors associated with these articles are shown in Table 2.

**Table 2.** Factors associated with the number of citations for articles on dentistry published by the top 10 publishers.

Top 12 editos	Statistics	Tcitations	Npages	PYears	NAuthors	Nreferencies
WILEY	Mean	16.5	8.2	5.9	5.4	35.2
	Estándar D.	26.7	2.7	3.6	2.7	16.4
(n=24125)	Min	0	1	0	1	1
Nonvitation (%)	Max	742	69	12	46	354
7.4	Med	9	8	6	5	33
ELSEVIER	Mean	18.6	7.2	5.8	5.8	33.2
	Estándar D.	29.7	2.8	3.4	3.3	16.7
(n=14790)	Min	0	2	0	1	1
Nonvitation (%)	Max	814	72	12	131	269
10.4	Med	9	7	5	5	31
QUINTESSENCE	Mean	11.8	8.3	6.3	4.7	32.2
PUBLISHING CO INC	Estándar D.	25.8	3.5	3.4	2.1	19.5
(n=14790)	Min	0	2	1	1	1
Nonvitation (%)	Max	1621	124	12	34	612
10.4	Med	6	8	6	5	30
CHURCHILL	Mean	12.6	5.8	6.4	5.3	24.0
LIVINGSTONE	Estándar D.	16.4	2.1	3.2	2.6	13.7
(n=6819)	Min	0	1	1	1	1
Nonvitation (%)	Max	475	24	12	61	196
6.8	Med	8	6	6	5	24
SPRINGER	Mean	9.5	8.1	4.0	5.4	33.7
	Estándar D.	23.1	2.5	3.1	2.4	15.0
(n=6741)	Min	0	2	0	1	1
Nonvitation (%)	Max	1407	34	12	30	255
15.5	Med	5	8	3	5	32
MOSBY-ELSEVIER	Mean	16.6	7.5	6.8	4.5	29.8
	Estándar D.	22.0	2.6	3.7	1.9	13.1
(n=5196)	Min	0	2	1	1	1
Nonvitation (%)	Max	332	29	12	21	174
6.0	Med	10	7	7	4	29
WB SAUNDERS CO-	Mean	13.0	8.6	6.7	4.4	28.6
ELSEVIER INC	Estándar D.	26.9	4.1	3.5	1.9	24.4
(n=4357)	Min	0	1	0	1	1
Nonvitation (%)	Max	1348	100	12	20	567
8.3	Med	7	8	7	4	24
SAGE PUBLICATIONS	Mean	23.2	7.4	5.1	6.6	31.6
INC	Estándar D.	45.9	1.8	3.9	3.8	11.5
(n=2656)	Min	0	2	0	1	1
Nonvitation (%)	Max	1040	21	12	38	204
13.3	Med	12	7	4	6	31
PERGAMON-ELSEVIER	Mean	13.1	7.7	6.1	5.9	39.6
SCIENCE LTD	Estándar D.	16.2	2.0	3.4	2.4	14.2
(n=2276)	Min	0	3	0	1	4
Nonvitation (%)	Max	219	22	12	20	162
5.0	Med	9	7	6	6	37
BMC	Mean	9.4	9.0	3.8	5.7	37.2
	Estándar D.	13.8	2.5	3.1	3.0	15.0
(n=2089)	Min	0	2	1	1	5
Nonvitation (%)	Max	152	44	12	37	179
15.8	Med	4	9	3	5	35
	ANOVA	136.03	616.8	420.39	286.59	382.64
	p	0.000	0.000	0.000	0.000	0.000

In addition, in the UK, for example, the UK Research and Innovation (UKRI) suggests that impact factors should not be used to indicate quality and should be ignored, as should other journal metrics. ^44^

In terms of the average number of citations, Sage Publications, Inc. stands out with 23.2 ± 45.9 citations and a maximum of 1,040; however, results show 13.6% noncitations.

Quintessence Publishing Co., Inc. shows the maximum number of citations (1,621), followed by Springer Nature, with 1,407 citations. However, the former also shows the second highest noncitation rate (15.5%) after BMC (15.8%) as well as the maximum number of pages (124 pages) and references (612) per article. The maximum number of authors was found in an article published by Elsevier (131 authors).

### Modelling the impact of scientific production on dentistry

On the one hand, Poisson’s model assumes that the mean is equal to the variance, but Table 1 indicates that the standard deviation (square root of the variance) is greater than the mean of the total number of citations per article. The presence of overdispersion is assumed to be evident. The presence of overdispersion was identified using the Lagrange multiplier test in the Poisson regression model (LR value = 15263275, p = 0.000 < 0.05) and confirmed by the likelihood ratio test in the negative binomial regression model (chibar2(01) = 8.8e+05, p = 0.000 < 0.05). The presentation of the results estimated for these models is omitted. However, these findings justify the discarding of Poisson’s regression model since resorting to alternative models was necessary.

On the other hand, according to Table 2, the percentages of noncitations for the articles published in the top 10 journals are within the range of 6.0-15.8%, suggesting a high occurrence of noncitations. Because the average number of citations is 9.4 citations per article, the probability of an article not being cited is 0.008%; this probability becomes very small (practically zero) as the average number of citations increases. In these circumstances, zero-inflated models are justified.

The zero-inflated negative binomial regression model and negative binomial hurdle regression model estimated for citations and noncitations are shown in Tables 3 and 4, respectively. The models do not show the results corresponding to the BMC publisher because that publisher constitutes the reference category; moreover, the regression coefficients establish the differences between BMC and the other publishers.

**Table 3.** Zero-inflated negative binomial regression model for citations of articles on dentistry in the top 10 journals.

TCitations		Coef.	Std.Err.	Z	P>z
**TCitations**					
Npages		0.036	0.002	21.120	0.000
Pyears		0.200	0.001	165.210	0.000
Nauthors		0.037	0.001	27.440	0.000
Nreferencies		0.011	0.000	38.160	0.000
TOP 10NPUBLISHER					
	Refence:	BMC			
WILEY		0.178	0.024	7.300	0.000
ELSEVIER		0.314	0.025	12.570	0.000
QUINTESSENCE PUBLISHING CO INC		-0.161	0.026	-6.120	0.000
CHURCHILL LIVINGSTONE		0.049	0.027	1.820	0.069
STRINGER		0.067	0.027	2.500	0.012
MOSBY-ELSEVIER		0.192	0.027	7.030	0.000
WB SAUNDERS CO-ELSEVIER INC		-0.129	0.028	-4.590	0.000
SAGE PUBLICATIONS INC		0.628	0.031	20.400	0.000
PERGAMON-ELSEVIER SCIENCE LTD		-0.060	0.031	-1.900	0.058
Constant		0.357	0.028	12.540	0.000
**inflate**					
Npages		-0.046	0.015	-2.990	0.003
Pyears		-1.843	0.053	-34.960	0.000
Nauthors		-0.079	0.013	-6.220	0.000
Nreferencies		-0.023	0.003	-7.430	0.000
TOP 10 PUBLISHER					
	Refence:	BMC			
WILEY		-0.951	0.126	-7.530	0.000
ELSEVIER		-0.285	0.131	-2.180	0.029
QUINTESSENCE PUBLISHING CO INC		0.636	0.138	4.600	0.000
CHURCHILL LIVINGSTONE		-0.522	0.174	-3.010	0.003
STRINGER		-0.798	0.136	-5.870	0.000
MOSBY-ELSEVIER		-0.877	0.194	-4-510	0.000
WB SAUNDERS CO-ELSEVIER INC		-0.335	0.191	-1.750	0.080
SAGE PUBLICATIONS INC		-0.601	0.157	-3.840	0.000
PERGAMON-ELSEVIER SCIENCE LTD		-0.678	0.287	-2.360	0.018
Constant		2.635	0.177	14.860	0.000
Analpha		-0.166	0.006	-28.540	0.000
Alpha		0.847	0.005		

**Table 4.** Negative binomial hurdle regression model for the number of articles on dentistry being cited in journals published by the top 10 publishers.

		Coef.	Std.Err.	Z	P>z
**Logit**					
Npages		-0.036	0.006	-5.680	0.000
Pyears		-0.513	0.007	-74.390	0.000
Nauthors		-0.096	0.006	-16.490	0.000
Nreferencies		-0.028	0.001	-22.710	0.000
TOP 10NPUBLISHER					
	Refence:	BMC			
WILEY		-0.433	0.070	-6.220	0.000
EL SEVIER		0.036	0.071	0.510	0.611
QUINTESSENCE PUBLISHING CO INC		0.495	0.075	6.560	0.000
CHURCHILL LIVINGSTONE		-0.525	0.085	-6.190	0.000
STRINGER		-0.143	0.074	-1.930	0.054
MOSBY-ELSEVIER		-0.573	0.091	-6.330	0.000
WB SAUNDERS CO-ELSEVIER INC		-0.155	0.089	-1.730	0.083
SAGE PUBLICATIONS INC		-0.130	0.091	-1.430	0.153
PERGAMON-ELSEVIER SCIENCE LTD		-0.467	0.121	-3.870	0.000
Constant		1.556	0.087	17.830	0.000
**negbinomial**					
Npages		0.036	0.002	20.010	0.000
Pyears		0.204	.0001	155.460	0.000
Nauthors		0.035	0.001	24.440	0.000
Nreferencies		0.010	0.000	34.000	0.000
TOP 10 PUBLISHER					
	Refence:	BMC			
WILEY		0.196	0.026	7.480	0.000
EL SEVIER		0.371	0.027	13.790	0.000
QUINTESSENCE PUBLISHING CO INC		-0.126	0.028	-4.440	0.000
CHURCHILL LIVINGSTONE		0.046	0.029	1.600	0.110
STRINGER		0.096	0.029	3.340	0.001
MOSBY-ELSEVIER		0.210	0.029	7.150	0.000
WB SAUNDERS CO-ELSEVIER INC		-0.127	0.030	-4.190	0.000
SAGE PUBLICATIONS INC		0.677	0.033	20.430	0.000
PERGAMON-ELSEVIER SCIENCE LTD		-0.061	0.034	-1.790	0.074
Constant		0.288	0.031	9.360	0.000
Analpha		-0.024	0.008	-2.950	0.003

With respect to citations, the negative binomial regression model ( Table 3) indicates that the number of pages (p < 0.001), the number of years since the article was published (p < 0.001), the number of authors (p < 0.001), and the number of references used (p < 0.001) contribute favourably to increasing the number of citations per article since the regression coefficients are positive. Regarding the publishers, in comparison with BMC, the publishers with the highest number of articles being cited were Wiley (p < 0.001), Elsevier (p < 0.001), Mosby-Elsevier (p < 0.001), and Sage Publications, Inc. (p < 0.001) but also Springer Nature (p < 0.05); moreover, those with the fewest citations were Quintessence Publishing Co., Inc. (p < 0.001) and Wb Saunders Co-Elsevier Inc. (p < 0.001). These results reflect that the factors have a favourable effect and that the differences between the publishers seeking to achieve a higher number of citations improved the h-index among dental researchers when adjusting for the other factors.

In reference to the zero-inflated model, that is, the model without citations, the factors under study contributed to the decrease in the number of publications, which is explained by the negative regression coefficient.

Some journals are known to limit the number of authors per article, especially when the topic covered in the articles does not justify the presence of numerous authors. Each publisher makes decisions in this regard.

The negative zero-inflated binomial regression model, on the one hand, confirms the presence of overdispersion by testing the likelihood ratio (chibar2(01) = 8.1e+05 and p = 0.000 < 0.05); on the other hand, the information criteria provide AIC = 531 580.4 and BIC = 531 848.5.

The hurdle regression model, which is presented in Table 4, also establishes the positive effect of these factors on the impact of scientific publications compared to the zero-inflated negative binomial regression model in both the direction and the magnitude of the effect. Likewise, the hurdle model confirms the differences in the number of article citations in the different journals compared to BMC; the model also shows where there are no differences. The similarity of the effect of the factors and the differences between the publishers are also maintained with respect to the percentage of citations, with the exception of the logistic model that did not show differences in the number of citations in Elsevier’s journals compared to those in of BMC (p > 0.05). The confirmation of the factors through this model shows that the factors under study are as important in evaluating both the number of citations and noncitations.

In the evaluation of the goodness of fit for the negative binomial regression model, Hurdle provides the values AIC = 531 390.6 and BIC = 531 658.8 as information criteria. It is important to note that when estimating this model, it is necessary to create artificial variables to represent the publishers.

Based on the assumption that a model with lower AIC and BIC values is better, Hurdle’s model is slightly more suitable than the negative zero-inflated binomial regression model; moreover, adducing a criterion of parsimony to decide otherwise may limit the choice of which model should be adopted in articles in dentistry. However, the findings show that both models will be useful when selecting robust factors associated with citations and noncitations in the dental field.

Between 2010 and 2021, there were differences among the publishers’ average output, according to ANOVA (F = 107.191, p = 0.000). Wiley was the publisher with the most publications (1,938 ± 256 articles/year), followed by Elsevier (1,221 ± 257 articles/year); there were significant differences between these major publishers and other publishers, according to Tukey’s test.

With a portfolio of 1,600 journals in various scientific categories, Wiley is the world’s leading publisher. Elsevier’s journals lead in the field of medicine and health sciences. ^45^ These articles published by these publishers differ in the average number of times they are cited (F = 136.03, p = 0.000) as well as in the number of pages (F = 616.8, p = 0.000), the number of years since publication (F = 420.39, p = 0.000), number of authors (F = 286.59, p = 0.000), and number of references used (F = 382.64, p = 0.000).

The publishers for which they articles show a lower percentage of noncitations than BMC were Wiley (p < 0.001), Springer Nature (p < 0.001), Mosby-Elsevier (p < 0.001), Sage Publications Inc. (p < 0.001), Elsevier (p < 0.05), Churchill Livingstone (p < 0.05), and Pergamon-Elsevier Science Ltd. (p < 0.05). In contrast, articles published by Quintessence Publishing Co., Inc., exhibit a higher percentage of noncitations (p < 0.001), a result that is adjusted for the effect of other factors.

### Phase 2 – Analysis of the association, evolution, and trends of the main studies

Based on the nature of the methodological design, results from Phase 2 are presented here. These results provide a rigorous analysis of the scientific output published by publishers; the results also show trends in the scientific output published by publishers within the time window selected for the extraction of articles and based on the factors associated with the impact of scientific production.

**Figure 3.**
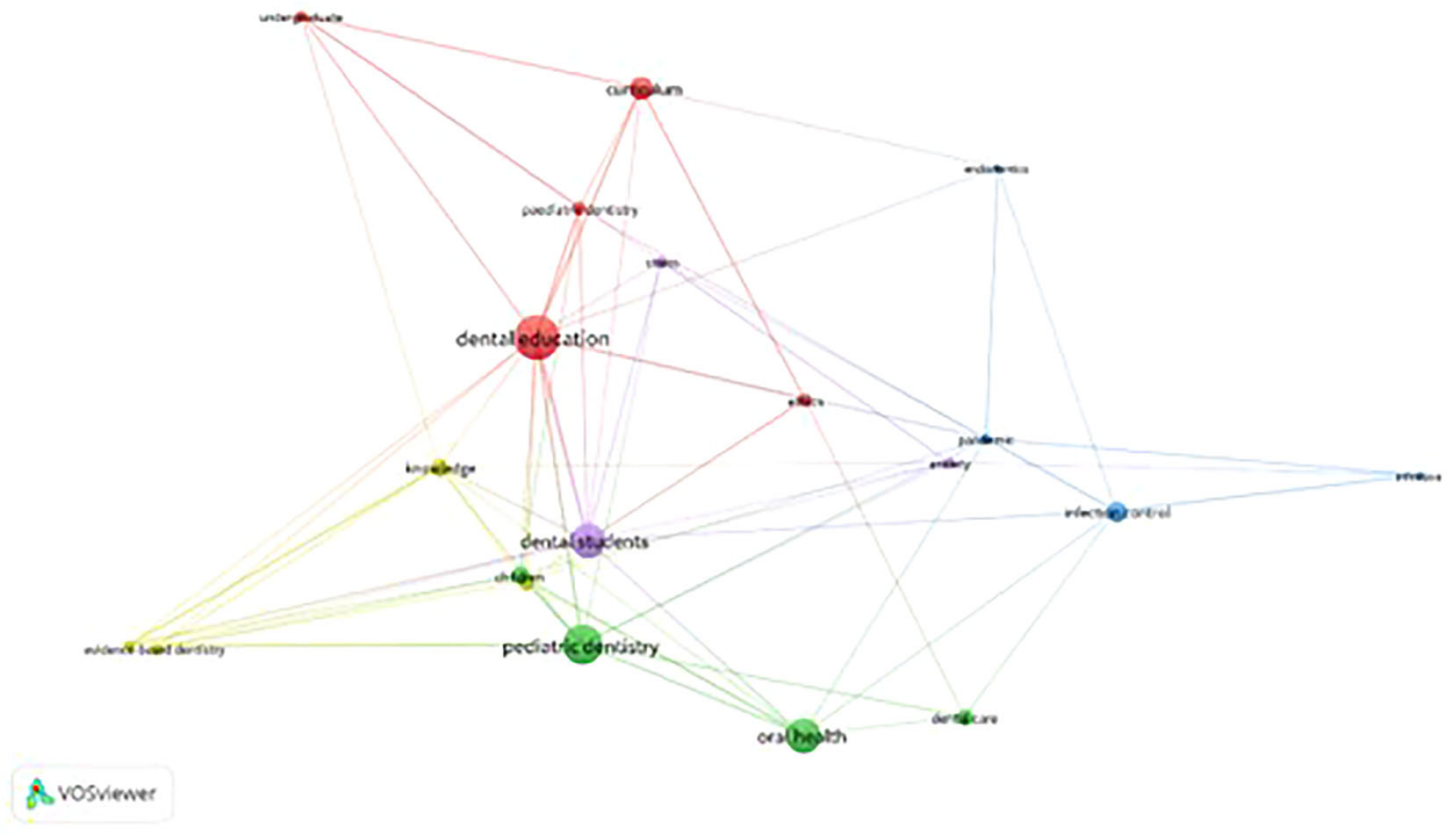
Grouping of thematic clusters. Figure 3 Alt Text. The Figure depicts the main keyword co-occurrence network in dentistry research from the WoS database, revealing thematic relationships and trends based on rigorous analysis of scientific output and impacts in Phase 2 of the methodological design.

The materialisation of the bibliometric analysis also allowed for an exhaustive analysis of the keywords associated with the scientific literature on dentistry so that the output covered in the literature, its associations, and the scientific trends represented can be understood. Therefore, Figure 3 presents the main keyword co-occurrence network in dentistry research that can be found in the WoS database; this figure allows us to identify relational and associative factors for keywords through clusters for an understanding of thematic affinities. ^46^

The main thematic cluster in red shows that “dental education” is a central concept. This concept is associated mainly with other keywords such as “paediatric dentistry”, “curriculum”, and “undergraduate”, which add important elements to the analysis; for example, it includes the idea of learning paediatric dentistry through cutting-edge educational models such as the flipped classroom ^47^ and virtual reality simulations. ^48^ Moreover, this approach allows for a rigorous analysis of the basic components that must be understood in the curricula of paediatric dentistry, as studied by Kaur *et al.* ^49^ in the Australian educational context.

The green thematic cluster is the second most relevant in terms of dentistry scientific production. On the one hand, results show that the concept of “paediatric dentistry” is also associated with other terms such as “children”, “oral health”, and “dental care”. These terms have generally been associated with shaping the scientific literature in terms of the dental needs of children who have special health needs, as described in studies that have analysed the dental characteristics of paediatric oncology patients, for example in Brazil. ^50^^,^^51^

On the other hand, there is an association between the green cluster and the yellow thematic cluster, which accounts for concepts such as “evidence-based dentistry” and “knowledge”.

Indeed, academia has expanded the knowledge, attitudes, behaviours, and practices of oral health professionals based on evidence-based dentistry. According to Rawat *et al.,* ^52^ this approach has improved the clinical experience of dental professionals. The approach has allowed researchers to improve their decision-making in relation to treatment safety; it has also increased treatments’ success rate. However, other authors have suggested that this success may be due to the development of robust and specialised educational programs that use evidence-based dentistry to allow dentists to improve their knowledge and skills. ^53^

In turn, this cluster also presents an important association with the purple thematic cluster, which accounts for overall essential concepts of health today, such as “stress” and “anxiety”; indeed, students of dentistry experience great psychoemotional burdens and high rates of anxiety. These concepts affect the dental system, including behaviours such as bruxism or cheek biting, which are important oral health problems. ^54^^,^^55^ This issue is of interest not only to dental students but also to general dentistry practitioners and specialists in paediatric dentistry, as shown in the context of Iran. ^56^

Finally, the blue thematic cluster includes important concepts such as “pandemic”, “infection control”, “endodontics”, and “infection”, which have become relevant in the scientific literature on dentistry in recent years. This cluster aids in the analysis of specific situations in the context of the COVID-19 pandemic; for example, it helps in assessing the risk mental health workers have had of contracting the virus, showing that it has been considerably low because of intensive triage and comprehensive preventive measures ^57^; moreover, the cluster shows the effectiveness of blended learning in dentistry, specifically in endodontics, in the pandemic-induced context of isolation. ^58^

**Figure 4.**
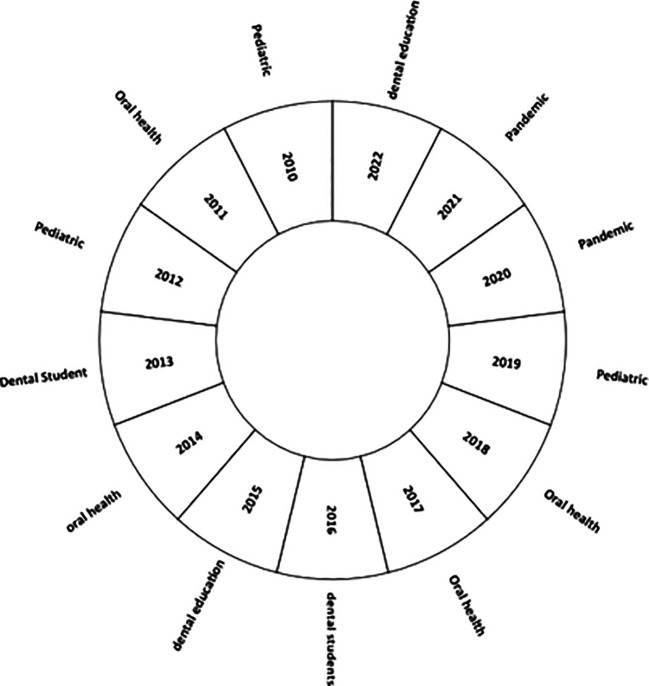
Evolution of scientific approaches. Figure 4 Alt Text. The figure showcases the conceptual evolution of dentistry's scientific literature, highlighting the most frequently explored keyword for each year within the specified database timeframe.

Subsequently, the analysis of the conceptual evolution of the scientific literature on dentistry is shown in Figure 4, which presents the most frequent and investigated keyword in each year during the time window included in the database. In that sense, it is clear that the trends in dentistry research at the beginning of the previous decade already revealed the interest of academics, researchers, and oral health professionals for paediatric dentistry, with “paediatric” being the most researched concept in 2010, time in which important advances were made on the topic. For example, in the study by Poletto and Junior, ^59^ one of the key review articles in the field, the authors concluded that the previous literature provided little conclusive scientific evidence, specifically in the Brazilian scientific literature, a context in which the prevalence of alveolar bone loss was also studied in children considered healthy and treated in private paediatric dentistry clinics. ^60^ Moreover, it is evident that this concept has dominated the literature since that year, constituting the most researched term in 2012 and 2019, which further indicates the relevance of the topic.

Moreover, dental education has been a central issue in the research field, and it was the most investigated concept for the year 2015, when important contributions were made. For example, Musa, Bernabé and Gallagher ^61^ studied the main motivations of Malaysian students for studying dentistry based on psychometric methodologies and the robust statistics of a confirmatory factor analysis. In addition, dental education has been the area of interest of the greatest number of studies in 2022, where the topic has been addressed in terms of state-of-the-art technologies, such as the implementation of standardised patient methodologies that allow students to develop communication skills, deeper diagnostic reasoning, and critical thinking. ^62^

In conjunction, it is evident that for 2020 and 2021, the priority in research was associated with the COVID-19 pandemic, and the term “pandemic” was the most studied concept in the scientific literature on dentistry. Some studies covered aspects associated with fears, eating habits, and perceptions of oral health, ^63^ and general evaluations of the impact of the pandemic on dentistry were made in specific contexts such as the Czech Republic, as evidenced in Schmidt *et al.* ^64^ These results contribute to the analysis of and debate on communication management during the current pandemic, a time during which dentistry has clearly emerged as a field in which research must be developed in consideration of international collaboration. The results for the last three years allow us to infer that the pandemic is an aspect that can propose new directions for research in this field.

**Figure 5.**
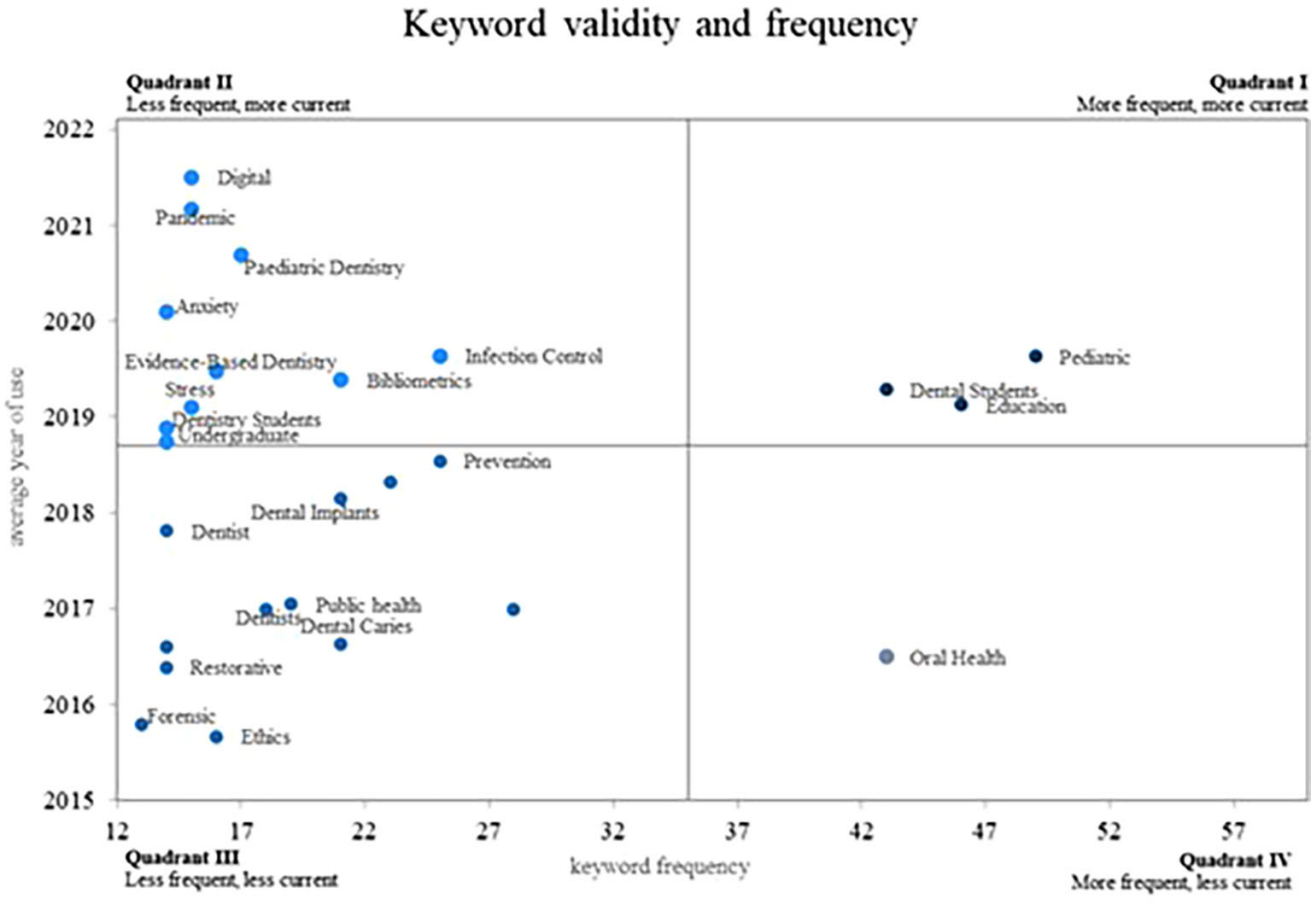
Validity and frequency of keywords. Figure 5 Alt Text. This figure displays a keyword analysis of indexed scientific activity in dentistry from the WoS database. Using a Cartesian plane, the figure illustrates the frequency of keywords against the year of usage, categorizing concepts into four quadrants based on frequency and recency. Quadrants I-IV depict varying combinations of frequency and currency in the scientific literature.

Finally, terms of keyword analysis for the scientific activity in dentistry that has been indexed in the WoS database are shown in Figure 5. From a Cartesian plane, this figure compares the frequency of keywords with the year in which they were used as a measure of validity. Therefore, there are a total of four quadrants: quadrant I includes the most frequent and current concepts; quadrant II plots the least frequent and most current concepts; quadrant III includes the least frequent and the least current concepts; and finally, quadrant IV shows the most frequent concepts that are less current in the scientific literature.

Therefore, quadrant IV, by positioning the concepts most frequently used for years showing a low average use, represents the terms whose use has declined in the research field; these terms only include “oral health”, which was previously key in stratifying the population for preventive dental care programs. ^65^

In a less prominent position, quadrant III groups the current concepts used the least frequently in the research field. This quadrant provides a general panorama for concepts that are not very prominent in the immediate future of dental research, noting terms such as “prevention”, “forensic”, “ethics”, “public health”, “dental caries”, and “restorative”. This phenomenon may indicate that researchers have already explored these topics in great depth or that there has been a constant evolution in the areas and/or the terms addressed; this scenario is reflected in the use of “forensic” in dentistry, which is currently related to the study of dental DNA, involving the use of prosthetic devices in recognition procedures, or to the issue of identification from the oral microbiota.

On the other hand, quadrant II includes those keywords that, although they are among the least frequently used in dentistry research, are among the most current, being categorised as emerging keywords. Among these main emerging keywords in the scientific literature on dentistry, there are research methodologies such as “bibliometrics”, which has been used in studies such as Paschoal *et al*.’s, ^66^ one of the most cited articles on the application of lasers in dentistry; also emerging is “infection control”, which in recent years was investigated by Kızılcı *et al*. ^67^ in relation to the level of knowledge that dental students should have regarding the factors that influenced the spread of the COVID-19 pandemic; and “digital” has also emerged, referring to the implementation of new technologies such as artificial intelligence in the dental context to improve medical diagnoses and patient care. ^68^ Emerging keywords are related to new technologies and new conditions, although they can also reflect the continuous development of an existing technology. The term “digital” can be associated with different concepts that involve not only artificial intelligence but automation, virtual reality and augmented reality, and 3D printing and robotic dentistry, among others.

Likewise, in terms of the specific analysis of the concept of the COVID-19 pandemic, future research must not explicitly focus on the analysis of that disease but understand that the post-pandemic context is already being discussed. Therefore, greater prominence will be given to the themes derived from this historical context, such as the application of new technologies, which were recently mentioned, and the response to the Industry 4.0 context or the Fourth Industrial Revolution.

Finally, quadrant I, which relates the most frequent and current use of concepts, is considered to account for the growing use of concepts on the subject; this quadrant includes the concepts that are the most prominent and relevant to the scientific literature on dentistry. It also includes the term “dental students”, who have been studied from different perspectives. For example, the effectiveness of e-learning classes in contexts such as Pakistan has been analysed ^69^ in a study in which the authors drew conclusions about the level of dissatisfaction generally perceived among students, revealing problems related to the use of new technologies. In addition, the keyword “education” was identified, where important contributions to dental practice have been generated, as evidenced in studies that have evaluated the quality of the educational system’s services for dental students based on a validated psychometric model such as SERVQUAL and on variables such as educational sector, country, gender, and year. ^70^ Finally, the concept of “paediatric” has emerged, referring to paediatric dentistry in which new approaches have made it possible to understand the role of new technologies such as cone-beam computed tomography; studying factors, including the degree of awareness; and knowledge of correct use. ^71^ The scientific production centered on students is focused on their skills and experiences that contribute to problem solving and lifelong learning; this research has focused on students’ performance in building meaning based on previous experiences and new information.

## Discussion and conclusions

Currently, a commercial strategic reorientation is taking place among the main academic publishers regarding a more democratic access to knowledge through open-access and alternative payment structures. However, an increase in the control of the academic infrastructure, which has been geared towards generating income, can strengthen the power of publishing houses, causing some researchers or institutions to be marginalised. ^72^

According to Posada and Chen, ^72^ some of the largest academic publishers use a set of elements, resources, or necessary services that allows their development within the publishing industry, which can generate a dependency known as oligopoly. ^72^^,^^73^ Such is the case for Taylor & Francis, one of the main academic publishers globally that engaged in content acquisitions between 2013 and 2015. However, according to our results, this trend does not appear to be relevant for the 10 main publishers during the 2010-2021 period in terms of scientific production in dentistry. ^74^

With respect to Elsevier and Wiley, the disproportionate ownership is evident in 2013, the year in which together with Springer, Taylor & Francis and Sage monopolised more than half of the total articles published. ^72^ According to the findings of the present study, although the line showing Wiley’s activities is concave in each time period, during the first period (2010-2016), Wiley exhibited essentially stable production with no linear (p = 0.211 > 0.05) or quadratic trends (p = 0.211 > 0.05). However, during the second period, the linear trend increased (p = 0.029 < 0.05), and the quadratic trend was not evident (p = 0.690 > 0.05), R2 = 92.7%.

Elsevier, which has integrated new products vertically into the life cycle of academic knowledge production, has created greater dependence on its products by offering greater convenience and by integrating these products into the decision-making process in universities; these universities must be up to date or improve their situation by acquiring Elsevier’s services. Elsevier generates income by owning data and, as a result, by promoting high-impact journals, a practice in which Wiley and Francis & Taylor have also engaged. ^72^ According to our findings, the curve showing Elsevier’s behaviour is concave–convex, similar to that of the WoS, with a discontinuity of 550 articles/year (t = 2.85, p = 0.029) and R2 = 94.3%. The data shows that Quintessence Publishing slightly changed its behaviour during the 2015-2022 period compared to the 2010-2014 period, with R2 = 83.8%. In this regard, a linear model would have exhibited an increasing annual trend of approximately 13 articles/year (t = 4.11, p = 0.002 < 0.05) and R2 = 62.9%.

The number of studies published in recent decades shows has trended upward. For example, five publishers accounted for more than 20% in 1973, increasing to 30% in 1996, 50% in 2006, and 53% in 2013. In 2013, 47% of all articles were distributed by three publishers. The production of the publishing house Reed-Elsevier has increased 1.5-fold since 1990, accounting for 24.1%. The production of the Springer Nature publishing house increased 2.9-fold, accounting for 11.9% of articles, and that of the publishing house Wiley-Blackwell increased 2.2-fold, accounting for 11.3%. In contrast, the number of publications by the American Chemical Society decreased by 5%, accounting for 3.4%; finally, the production of the Taylor & Francis publishing house increased 4.9-fold, reaching 2.9% of all publications. ^47^

Luchilo ^75^ indicated that for the year 2015, in the publishing market, the four leading publishers concentrated a considerable number of journals; these publishers are Springer Nature (2,987 journals), Elsevier (3,057 journals), Wiley (2,339 journals), and Taylor & Francis (2,105 journals). According to the present study, among the 10 publishers with the highest number of publications in terms of dentistry scientific output, 333 were found in Springer Nature, 1,072 were found in Elsevier, and 1,847 were found in Wiley; the number of publications for Taylor and Francis was not included in the table.

Fereira *et al.* ^76^ reviewed the output included in dentistry journals in the 2015 edition of the Journal Citation Reports in the category “Dentistry, Oral Surgery & Medicine”. Among the publishing houses that support these journals, the American publisher Wiley-Blackwell was the most represented, controlling 24 of the 91 journals identified, followed by Elsevier with 10 journals and Quintessence Publishing Co. with the same number. These results coincide with those found in the present investigation in terms of scientific production; indeed, between 2010 and 2022 (August), Wiley hosted 24,125 publications, Elsevier hosted 14,790, and Quintessence Publishing Co., Inc., hosted 7,592. Likewise, Luengo’s results showed that, out of the 10 main journals publishing research in dentistry, Wiley-Blackwell published five, Elsevier Science published four, and Sage Publications published one.

The results of the bibliometric analysis on dental caries, which is one of the most prevalent oral diseases worldwide, ^77^ with abundant research addressing this issue in various fields of dentistry, developed from the analysis of academic production from 2014 to 2018 in the WoS. This issue has shown to follow a linear growth pattern with a low slope, suggesting a near saturation point. ^78^ Moreover, the analysis carried out on the same database regarding scientific production in dentistry coincides with the present research because it shows a concave growth curve during the first period of 2010-2016. However, during the second period, 2017-2021, the curve is shown to be convex, which means that the topic is not approaching a saturation point.

Based on the results obtained in the present study, it is important to consider specific aspects, such as those studied by Li *et al.,* ^79^ who analysed the format in the structure and the policy pertaining to the relevant publishing houses in relation to the selection of articles in an easy, rapid way that is more relevant to readers, hence promoting a more efficient indexing of research. Compared to a highly structured format, the Introduction, Methods, Results, and Discussion (IMRandD) format was used more frequently and was required to be used in the abstracts of randomised controlled trials (RCTs) and systematic reviews (SRs) in the field of dentistry.

The factors associated with the citations of scientific production in dentistry indexed in Scopus suggest some explanatory variables, such as the aspects related to the evolution of scientific production by the editor. Within these aspects, the number of pages, authors and references used were important. Moreover, the analysis of associations, and the evolution and trends in the main studies include the study of the association of thematic groups, the evolution of scientific approaches, and the validity and frequency of keywords. These studies provide evidence on which the scientific community can focus in relation to dentistry to increase its scientific production by concentrating on strategies that generate good results.

### Strengths and limitations

A total of 110,269 articles published in WoS journals were reviewed. Although this volume is large, it must be recognised that this study constitutes only a partial vision because other databases have not been included. On the one hand, the analyses carried out include different numbers of articles, which could confuse readers; however, it was strictly necessary, as the annual scientific production selected could not include the year 2022, which is not published until the third of that month. The factor analysis did include all of the studies, and it was evident that the 9,034 articles published in 2022 contained current bibliometric information. On the other hand, comparing journals was expected for the analysis of impact factors; however, editorials were considered for technical reasons, and the journals were part of more than one scientific category. Moreover, when a good article was not accepted in a journal based on field or category, suggestions for other journals were received from the same publisher. Finally, the bibliometric analysis includes 1,000 more recent articles because these contain information on emerging issues from the point of view of journals and therefore publishers. Perhaps these results constitute valid reasons for the authors, but not for the readers.

The study includes modelling in the bibliometric analysis. First, for a demonstration through statistical tests rather than subjective appraisals, the great growth in scientific production in dentistry is shown through discontinuous regression models. Changes in the timing of production are shown through regression models by sections, and these models are adaptable to biometric analyses on emerging issues in various scientific areas or categories. Moreover, this study retrieves factors related to the positive impact (citations) or lack thereof (noncitation) of articles published in dentistry. Authors and journals may take these results into consideration to improve their bibliometric metrics and thereby increase the prestige of the editors while guiding them in making decisions about editorial policies. Finally, bibliometric analysis provides information on emerging issues that authors should consider regarding the acceptance of their articles in WoS journals using classical and current methodologies.

Additionally, although it is true that the factors associated with the citations of scientific production in dentistry are similar to those used for other health disciplines, we recognise that some factors can be differentiated, such as those base research topics, since dentistry focuses on diagnosis, the treatment and prevention of diseases and the conditions of the dental system. Other health disciplines may focus on different areas of the human body or methodological aspects of research. Although there is a wide variety of research methodologies addressed in health disciplines, some may be more common in dentistry. The existence of specialised magazines that publish scientific articles have framed dentistry. Moreover, some health disciplines have superior means of dissemination for research results compared to dentistry, which affects the output in that field.

## Conclusions

Our results show that the trend in the dentistry scientific output published by the main publishers is discontinuous in regression models. The main factors associated with the citation and noncitation of scientific output in dentistry are shown to be the editors working at these journals, the number of pages in the articles, the year of publication, the number of authors and the number of references. in the article, according to zero-inflated negative binomial regression and Hurdle models. The main clusters that define the conceptual associativity on dentistry are the red and green clusters, where the red cluster puts in relation concepts associated with the design of university curricula in paediatric dentistry, and the green cluster puts in relation elements associated with oral health in children. The thematic evolution in the scientific literature on dentistry shows that there have been changes since the first investigations on paediatric oral health; indeed, investigations in recent years have focused on the analysis of the subject during the pandemic period and on the importance of dental education. Finally, the main keywords that have been increasingly used within the scientific body in dentistry are “dental students”, “education”, and “paediatrics”, while the top emerging keywords are “digital”, “pandemic”, “anxiety”, and “infection control”. Further research in this important area is needed.

## Data Availability

Zenodo: Factors Associated with Scientific Production Citations in Dentistry: Zero-inflated Negative Binomial Regression and Hurdle Modelling, https://doi.org/10.5281/zenodo.8387311. ^80^ Data are available under the terms of the Creative Commons Attribution 4.0 International license (CC-BY 4.0).
