# Supplementary material for: Factors associated with scientific production citations in dentistry: Zero-inflated negative binomial regression and hurdle modelling
Source: F1000Res. 2023 Oct 12;12:1321. [Version 1] doi: 10.12688/f1000research.141422.1 (PMC11226947; doi:10.12688/f1000research.141422.1)
Supplement: Peer review history

# Reviewer report: Reviewer response for version 1

**DOI:** 10.5256/f1000research.154866.r293804

**Published:** 2024-07-05

**Authors:** Brenda Yuliana Herrera Serna

**Affiliations:** 1 Universidad Autonoma de Manizales, Manizales, Caldas, Colombia

Thank you for the manuscript. It is interesting and provides an enriching overview. Given the broad search strategy, the volume of findings is to be expected. The statistical analysis is relevant and shows the application of diverse tools for the practice of dental research and education.  Although WOS is a comprehensive database, it would be appropriate to state why it is the only one used. There are many other data that are extracted from this type of analysis, such as the most productive countries, institutions and researchers. The 10 most cited articles is also an important source of information. These are only suggestions, and do not interfere with the review.

Is the work clearly and accurately presented and does it cite the current literature?

Yes

If applicable, is the statistical analysis and its interpretation appropriate?

Yes

Are all the source data underlying the results available to ensure full reproducibility?

Yes

Is the study design appropriate and is the work technically sound?

Yes

Are the conclusions drawn adequately supported by the results?

Yes

Are sufficient details of methods and analysis provided to allow replication by others?

Yes

Reviewer Expertise:

Oral epidemiology

I confirm that I have read this submission and believe that I have an appropriate level of expertise to confirm that it is of an acceptable scientific standard.

# Reviewer report: Reviewer response for version 1

**DOI:** 10.5256/f1000research.154866.r235646

**Published:** 2024-03-25

**Authors:** Carolina Caleza Jimenez

**Affiliations:** 1 University of Seville, Seville, Spain

This article is very interesting. The data analysis is very complete with two phases, with sufficient details and adequate statistical analysis.

 It would be interesting to highlight the role of universities in providing publications to students or young researchers. The conclusions respond to the questions formulated to achieve the objectives, although they should be more cautious in the statements.

Is the work clearly and accurately presented and does it cite the current literature?

Yes

If applicable, is the statistical analysis and its interpretation appropriate?

Yes

Are all the source data underlying the results available to ensure full reproducibility?

Yes

Is the study design appropriate and is the work technically sound?

Yes

Are the conclusions drawn adequately supported by the results?

Partly

Are sufficient details of methods and analysis provided to allow replication by others?

Yes

Reviewer Expertise:

Paediatric dentistry

I confirm that I have read this submission and believe that I have an appropriate level of expertise to confirm that it is of an acceptable scientific standard.

## References

[1] : Predatory publishing in medical education: a rapid scoping review. *BMC Med Educ* .2024;24(1) : 10.1186/s12909-024-05024-x 33 10.1186/s12909-024-05024-x 38183007 PMC10770935
